# Forty years since Alma-Ata: do we need a new model for noncommunicable diseases?

**DOI:** 10.7189/jogh.09.010316

**Published:** 2019-06

**Authors:** David Beran, Pablo Perel, J Jaime Miranda

**Affiliations:** 1 Division of Tropical and Humnitarian Medicine, University of Geneva and Geneva University Hospitals, Geneva, Switzerland; 2 London School of Hygiene and Tropical Medicine, London, United Kingdom; 3 World Heart Federation, Geneva, Switzerland; 4 CRONICAS Centre of Excellence in Chronic Diseases, Universidad Peruana Cayetano Heredia, Lima, Peru; 5 School of Medicine, Universidad Peruana Cayetano Heredia, Lima, Peru

Last year marked the 40th Anniversary of the Alma-Ata Declaration which stated that Primary Health Care (PHC) was “essential health care based on practical, scientifically sound and socially acceptable methods and technology made universally accessible to individuals and families in the community.” [1] The Declaration mentioned the need for PHC to evolve with changing disease and socio-economic conditions, focus on the main health problems by providing health promotion, prevention, care and rehabilitation, involving the community resources at large for health, empowering communities and adequate human resources.

Forty years ago, the main causes of morbidity and mortality were communicable diseases impacting children under the age of 5 and maternal mortality [2]. Responses focused on providing: growth monitoring (G); oral rehydration therapy for diarrhea (O); promotion of breastfeeding (B); immunizing children (I); family planning (F); food supplementation (F); and promoting female literacy (F) leading to the acronym GOBI FFF [3].

In 2018, the leading global cause of morbidity and mortality are Noncommunicable diseases (NCD) [4]. NCD targets are included in the Sustainable Development Goals (SDG) [5] and the importance of PHC in achieving the SDGs has been noted [6]. In addition the recent Declaration of Astana, marking the 40th Anniversary of Alma-Ata, stated that PHC is a cornerstone for universal health coverage (UHC) and the health-related SDGs [7]. Included in the Declaration of Astana is mention of NCDs with the importance of the 4 common risk factors (tobacco use, unhealthy diets, insufficient physical activity and the harmful use of alcohol) and PHC’s role in the prevention, control and management of these conditions. However, the text of the Declaration lacks concrete measures.

For NCDs, PHC needs to provide a wide range of preventative and curative services. This cuts across all ages, children with type 1 diabetes and adults with hypertension, and also needs a gender specific approach, for example with the management of gestational diabetes. NCDs require a variety of factors from the health system, access to medicines, diagnostics, education, continuity of care, in addition to a wide range of societal factors across the life course.

A model to address NCDs at PHC would therefore need to include Prevention (P) focused on the community at large as well as targeted to those with specific risk factors. For those without an NCD their risk profile needs to be assessed. For those with NCDs there is the need to look beyond their individual disease to focus on the individual and their wide range of needs, such as social, psychological and health. All these responses need to be adapted to the local context.

WHO’s Package of Essential Noncommunicable (PEN) [8] provides a framework for PHC in the different elements needed for Services (S) to be delivered and tools that are needed, including diagnostics and medicines. Clinical needs around continuity of care and multi-morbidity and wider elements of responsive services to people’s needs also need to be addressed. From an organizational perspective the management of people with an NCD has to consider that once the individual is diagnosed their NCD needs to be considered in each interaction with the health system. For example, a pregnant woman with hypertension requiring services for both her pregnancy and her NCD. There is also the need to manage acute exacerbations of the individuals NCD or acute health needs of an individual with an NCD. It is also important to see how PHC fits into the overall health system and community in providing the first point of entry for care, a link to higher levels of the health system or other services to improve health as well as a coordinating role with a focus on the individual. Technology is also an opportunity to facilitate the delivery of care at PHC.

The P for Prevention and the S for Services need to focus both on the Individual (I) and Community (C) as a whole recognizing that a community is made up of individuals with different ages, gender and health needs.

For NCDs there is the need to address a wide range of societal factors in communities (C) and adapt responses to this local context, as well as the life course of the individual (I). The individual will need to be emphasized and not specific diseases due to multi-morbidity, mental health and ageing being additional important considerations. This community will fall within a spectrum of risk from those at no risk; those with certain factors, such as being overweight or obese; those already with a physiological impact of a given risk factor (raised blood pressure); those with an undiagnosed NCD; those already with an NCD and those with an NCD related complication. All these sub-populations will require a tailored response for their current needs and risks.

**Figure 1:**
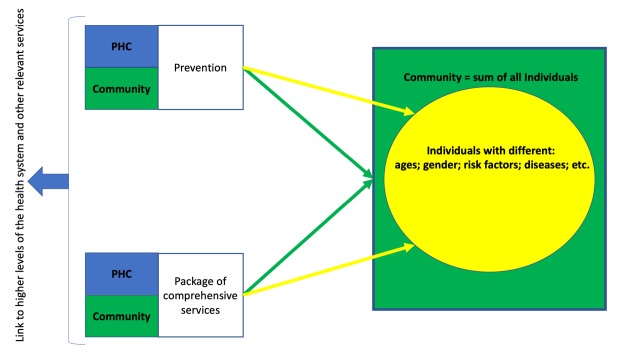
Prevention, Services, Individual and Community (PSIC) Model

PHC needs to be embedded within communities and seen as the link between populations and their health system. Community also includes the role of caregivers in providing support beyond the formal health system and aligns with the WHO’s view of developing people centered care with “people hav[ing] equal access to quality health services that are co-produced in a way that meets their life course needs, are coordinated across the continuum of care, and are comprehensive, safe, effective, timely, efficient and acceptable” [9]. These elements together form the proposed PSIC model (**Figure 1**) which will benefit from effective use of different cadres of health personnel and redefining the roles they play.

**Figure:**
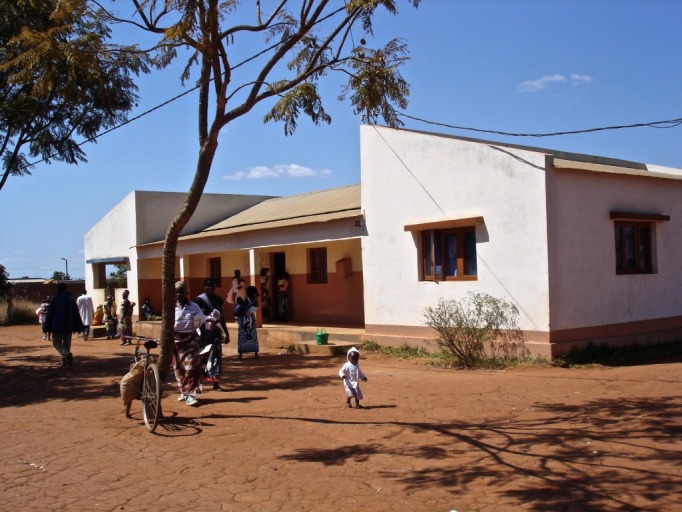
Photo: from David Beran’s collection (used with permission).

GOBI FFF provided a set of “simple” interventions for PHC to deliver, which targeted only women and children, and often required only one interaction with the health system, eg, delivery of a vaccine. The PSIC model targets a wider population, with a life-course approach needed as well as continuous delivery of services for all health needs. In order achieve the goals stated in the SDGs and WHO NCD Global Action Plan. PHC responses need to be developed. The 40 years of the Alma-Ata Declaration and PSIC model offer a unique opportunity to do this. The roles communities play in defining the solutions that the health system needs to provide is essential [10] and PHC provides a unique platform to do this and the PSIC a framework on which to build this.
